# Simulation of the Melt Conveying Zone of a Single-Screw Extruder for Mixed Polymer Materials Using an Isothermal Analytical Flat Plate Model

**DOI:** 10.3390/polym17233145

**Published:** 2025-11-26

**Authors:** Emil Wagner, Christian Kneidinger, Christoph Burgstaller, Gernot Zitzenbacher

**Affiliations:** 1 School of Engineering, University of Applied Sciences Upper Austria, Stelzhamerstr. 23, 4600 Wels, Austria; christian.kneidinger@fh-wels.at (C.K.); christoph.burgstaller@tckt.at (C.B.); g.zitzenbacher@fh-wels.at (G.Z.); 2 Transfercenter für Kunststofftechnik GmbH, Franz-Fritsch-Str. 11, 4600 Wels, Austria

**Keywords:** polymer recycling, predictive modeling, rheological properties, single-screw extrusion, melt conveying zone, polypropylene, polyamide 12, capillary rheometry, blends

## Abstract

An optimized extrusion process is desired for both an environmentally friendly and economically sustainable recycling process. The aim of this study is to simulate the melt conveying zone of a single-screw extruder when using contaminated polymers instead of commonly used pure materials, to optimize a mechanical recycling process, and to reduce the number of measurements needed for rheological input data by using mixing rules. Polypropylene (PP) is blended with a polyamide 12 (PA 12) grade and another PP grade to introduce polymer impurities into the material. The blends are subjected to extrusion experiments in a lab-scale single-screw extruder with pressure and temperature sensors along the barrel. An isothermal analytical simulation model is proposed using representative shear rate values and rheological mixing rules to calculate the pressure distribution along the screw channel throughout the melt conveying zone. The rheological input data for the simulation is taken from high-pressure capillary rheometric measurements, but also substituted with values derived from mixing rules. The results show that the application of the shear viscosity through mixing models yields simulated pressure values similar to those measured in the experiments. With the introduction of representative viscosity into the model, relative deviations of around 5% at certain screw speeds can be achieved.

## 1. Introduction

In accordance with European Union Directive 94/62/EC, the amount of recycled plastics used for new packaging must be increased to 55% by the end of 2030 [1]. To achieve that goal, an increase in the mechanical recycling of polymers is needed. A mandatory part of mechanical recycling processes for polymers is the extrusion step, such as the commonly used single-screw extrusion [2]. Here, bulk polymer is fed from a hopper into a single rotating screw within a heated barrel, where the material is compacted, compressed, melted, conveyed, and mixed, and finally extruded through a shaped die to create the desired shape of a continuous product, like a film for packaging [3,4].

The extrusion and material behavior of virgin or pure materials are widely studied topics [5]. However, the feedstock supplied to mechanical recycling rarely consists of such homogenous polymers [6,7]. This is because a large amount of recollected polymer waste comes from packaging films [8,9], which consist of multiple layers of different polymers [10], as this is required to achieve the needed barrier properties for food packaging [11] to counteract the diffusion of unwanted gases [12,13] into the product. As such, the properties of materials used as feedstock in mechanical recycling are different from virgin materials, not only in terms of mechanical properties [14,15,16] but also regarding their rheological behavior [17,18]. The latter, in particular, can affect the extrusion step of the recycling process, as changes in viscosity may impact the quality of the final product [5,19].

A commonly used method for measuring the viscosity of polymers under conditions similar to extrusion is high-pressure capillary rheometry [20,21]. Here, molten polymer is pushed through a capillary of set dimensions by a piston moving at a fixed speed, resulting in specified shear rates. Measurements with pressure transducers allow calculation of the wall shear stress in the capillary and, consequently, calculation of the shear viscosity after applying mathematical corrections for shear stress [22] and shear rate [23]. As these capillary rheometry tests would take too long to perform for every single feedstock, an alternative solution using mixing rules was suggested [24]. Using mixing rules such as those proposed by Bingham [25] or Heitmiller [26], Arhenius [27] or Bersted [28], Tsenoglou [29], and Kendall and Monroe [30], as well as a linear mixing model, it was shown that approximating measured viscosity data with appropriate mixing rules yielded results within a small margin of error for miscible blends of two polypropylene (PP) grades and immiscible blends of PP with polyamide 12 (PA 12) [24].

The central extrusion step is critical to the financial viability of mechanical recycling, as it is very energy-consuming [31]. Therefore, optimizing single-screw extrusion using polymer mixtures, which are common in recycling materials, is important. A typical single-screw extruder is divided into multiple functional zones, such as the solids conveying zone, the melting zone, and the melt conveying zone [4,19]. Various methods for simulating these zones have been formulated in the past. For example, the melting of polymer mixtures in single-screw extruders has been analyzed in previous studies using model experiments [32,33]. Finite-difference modeling of the melt conveying zone, especially considering the cross-sectional flow, residence time, and deformation, was described by Zitzenbacher [34] for pure polymer materials.

In this study, an isothermal analytical flat plate model for the simulation of the melt conveying zone of a single-screw extruder used for mechanical recycling with a focus on mixed polymer materials is presented. For this purpose, the viscosity curves of the polymer mixtures are calculated using mixing rules based on rheological data of the pure materials and incorporated into the calculation model. Two different calculation models, a basic flat plate model and an enhanced representative model, are proposed, and the simulation results are validated through lab-scale extrusion experiments. The intent is to show that proper simulation of functional extruder zones is possible while using input data from mixtures rather than commonly used pure materials.

## 2. Materials and Methods

### 2.1. Production of Polymer Blends

First, polymer mixtures made from two types of PP (HB600TF (PP_1_) and HD234CF (PP_2_); both Borealis AG, Vienna, Austria) and a PA12 type (Grilamid L25 (PA12); EMS-CHEMIE HOLDING AG, Herrliberg, Switzerland) were produced using a co-rotating twin-screw extruder (Leistritz ZSE 27 Maxx; Leistritz Group, Nuremberg, Germany) equipped with separate dosing units (Congrav OP5 and Congrav M dosing; Kubota Brabender Technologie GmbH, Duisburg, Germany) and a strand pelletizer. For better miscibility of the PP/PA12 blends, a PP-based coupling agent (Orevac CA100 (PPgMAH); Arkema Functional Polyolefines, Colombes Cedex, France) was introduced to the base PP_1_ at a weight fraction of 4 wt%. The polymers were mixed so that one of the PP types served as the base polymer, while the added PP and PA12 types served as impurities similar to the contaminants found in polymer recycling. All the created mixtures and the respective weight fractions of the polymers used are shown in Table 1.

### 2.2. Acquisition of Input Data for the Simulation

#### 2.2.1. Material Data

To obtain the shear viscosity data needed for the simulation of the melt conveying zone, high-pressure capillary rheometry was performed on the mixtures listed in Table 1. A high-pressure capillary rheometer, Rheograph 6000 (Göttfert, Buchen, Germany), was used for the tests, which were conducted at temperatures of 200 °C and 230 °C for the PP_1_/PP_2_ mixtures and 230 °C and 250 °C for the PP_1_/PA12 mixtures. The measured apparent values were corrected using the Bagley correction [22] and the Weißenberg Rabinowitsch correction [23] to achieve true values of the wall shear stress *τ* and the shear rate $\dot{\gamma }\;$, respectively. Next, the true viscosity values η for each mixture at both measurement temperatures were fitted to a single curve using a simplified version of the Bird–Carreau–Yasuda rheological model [35], together with a temperature shift factor $a_{T}$, according to Arrhenius [36], as seen in Equation (1):

(1) $$\eta \left(\dot{\gamma },T\right)\;={\;a}_{T}\left(\eta_{0}{\left(1+{\left|a_{T}B\dot{\gamma }\right|}^{a}\right)}^{\frac{\left(n-1\right)}{a}}\right)\;with\;a_{T}=e^{\frac{E_{0}}{R}\left(\frac{1}{T}-\frac{1}{T_{B}}\right)},$$

where $\eta_{0}$ is the shear viscosity at zero shear rate, *B* is the reciprocal value of the shear rate at the slope of the Newtonian plateau, *a* is a material-specific modeling parameter, *n* is the power-law exponent, $E_{0}$ is the activation energy, and $T_{B}$ is the reference temperature for the shift factor. Here, a numerical solver (generalized reduced gradient nonlinear solver; Microsoft Excel 365, Microsoft Corporation, Redmond, WA, USA) was employed in combination with the least-squares method to calculate $\eta_{0}$, *B*, *a*, *n*, $E_{0}$ to fit a temperature-independent shear viscosity curve for every mixture. The solver was repeatedly used until no further decrease in the deviation of the fits to the measured data could be observed.

As an alternative to computing the fits from measured data, an additional approach of using mixing models to calculate viscosity values for the mixtures $\eta_{M}$ (by only using measurement data of the pure materials) was applied. For the PP_1_ and PP_2_ mixtures, a linear mixing model was employed in Equation (2), while for the PP_1_ and PA12 mixtures, the model proposed by Bingham and Heitmiller [25,26] was used, Equation (3), as these were found to be the most accurate for miscible or immiscible blends, respectively [24]:

(2) $$\eta_{M_{lin}}=\eta_{A}w_{A}+\eta_{B}w_{B},$$

where $w_{A}$ and $w_{B}$ are the weight fractions of the components and

(3) $$\eta_{M_{B/H}}=\frac{1}{\frac{v_{A}}{\eta_{A}}+\frac{1-v_{A}}{\eta_{B}}},$$

where $v_{A}$ and $v_{B}$ are the volume fractions of the components. From here, the temperature-independent fits were generated identically as before, using Equation (1) and a numerical solver. The measurement data used for the calculation of the fits are listed in Table A1, Table A2, Table A3, Table A4, Table A5 and Table A6 in Appendix A.

#### 2.2.2. Extrusion Experiments

To describe the accuracy of the simulations using the input data created from mixing rules, the previously created mixtures were subjected to an extrusion experiment in a lab-scale smooth-bore single-screw extruder (Collin E20M, COLLIN Lab & Pilot Solutions GmbH, Maitenbeth, Germany). The extruder with an L/D ratio of 25 D with a diameter *D* of 20 mm was equipped with pressure and mass temperature sensors along the barrel located at the positions 8 D, 16 D, and 24 D. Furthermore, a single-flighted, single-pitched three-zone screw was employed, which incidentally had its melt conveying zone located between 16 D and 25 D, largely aligning with the last two pressure sensors, providing good information on the pressure loss from the beginning to the end of the zone. The geometric data for the screw are presented in Table 2.

**Figure 1:**
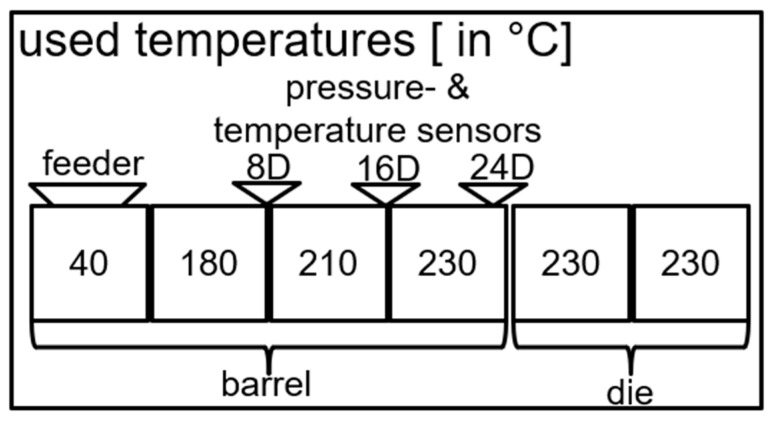
Schematic depiction of the extruder with the temperature settings used in the extrusion experiments alongside the locations of the pressure and temperature sensors. The melt conveying zone of the screw is located entirely within the last section of the barrel of the extruder.

A slit die with a die width of 50 mm was set at the tip of the extruder. The mixtures were then extruded at 60, 120, and 180 rotations per minute for 15 min each, using the temperature profile depicted in Figure 1. During this time, the pressure measured by the transducers and the temperature of the melt measured by the temperature sensors were recorded. The mass throughput of the extruder was determined by weighing the extrudate extruded over one minute for a total of five measurements per mixture, from which the average was taken. The melt density of the mixtures was derived from melt volume rate measurements (Zwick Roell MFlow, ZwickRoell GmbH & Co. KG, Ulm, Germany) performed at the same temperature as the extrusion experiments. The mass throughputs, in conjunction with melt density values at the extrusion temperatures, were used to calculate the volumetric throughput of each mixture with the processing parameters used in the experiment.

### 2.3. Simulation of the Melt Conveying Zone

**Figure 2:**
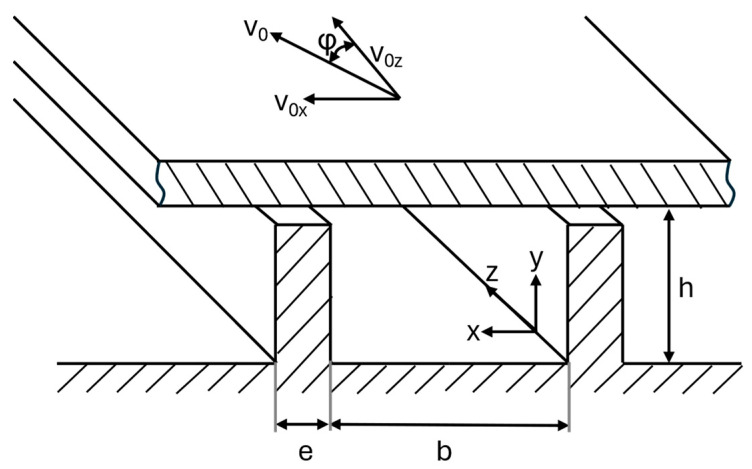
Schematic depiction of the flat plate model, which assumes that the helical screw channel is treated like a rectangular channel with height *h*, width *b* and flight width *e*. *φ* is the pitch angle and *v*_0_ is the flow velocity in the various directions. The figure was redrawn based on Rauwendaal [3].

To simplify the simulation of melt flow in the melt conveying zone, various assumptions are made based on the flat plate model, as described by Rauwendaal (Figure 2) [3]. First, a steady flow front is assumed. Furthermore, initially, the fluid is considered Newtonian, and wall effects caused by the flight flanks of the screw are disregarded. To address the shear thinning behavior of polymers, wall effects, and leakage flow across the flights, correction factors are introduced into the calculation of the volume flow through the melt conveying zone in a later step.

The flow velocity of the melt *v_0_* is defined as

*v*_0_ = *D*
*π*
*N*, (4)

where *D* is the diameter of the screw and *N* is the rotation of the screw. Introducing the pitch angle *φ* into the equation yields the melt velocity in the channel direction *v*_0*z*_ through

*v*_0z_ = *D*
*π*
*N*
*cosφ* (5)

and the melt velocity of the perpendicular flow *v*_0*x*_ through

*v*_0*x*_ = *D*
*π*
*N*
*sinφ*. (6)

The mean shear rate $\bar{\dot{\gamma }}$ within the channel can then be computed with the channel depth *h* as

(7) $$\bar{\dot{\gamma }}=v_{0}/h.$$

Next, the total volumetric throughput $\dot{V}$ is defined as the superposition of the drag- and pressure-induced flow

(8) $$\dot{V}={\dot{V}}_{d}-{\dot{V}}_{p},$$

with the drag-induced flow ${\dot{V}}_{d}\;$ as

(9) $${\dot{V}}_{d}=\frac{v_{0z}ibh}{2}$$

and the pressure-induced flow ${\dot{V}}_{p}$ as

(10) $${\dot{V}}_{p}=\frac{ibh^{3}}{12\eta }\frac{\Delta p}{Z},$$

where *i* is the number of flights of the screw, *b* is the width of the channel, Δp is the pressure change along a set segment of the metering zone, and *Z* is the pitch.

At this point, the previously mentioned correction factors are implemented into the equation. First, the factors *f_d_* and *f_p_*, which correct wall effects arising from polymer contact with the flight flanks, are used as proposed by Hensen, Knappe, and Potente [19]:

(11) $$f_{d}=0.1342{\left(\frac{h}{b}\right)}^{2}-0.6412\frac{h}{b}+1.0115$$

and

(12) $$f_{p}=0.1632{\left(\frac{h}{b}\right)}^{2}-0.7503\frac{h}{b}+1.0143.$$

The shear-thinning flow behavior of polymers can be considered by implementing the correction factors $f_{\eta d}$ and $f_{\eta p}$, as suggested by Rauwendaal [3] using the flow exponent *n*:

(13) $$f_{\eta d}=\frac{4+n}{5}$$

and

(14) $$f_{\eta p}=\frac{3}{1+2n}.$$

Finally, there is always a small leakage flow across the flight flanks, which affects the total volumetric throughput of the extruder. Again, the correction factors $f_{Ld}$ and $f_{Lp}$, taken from Hensen, Knappe, and Potente [19], are calculated for the drag-induced flow as

(15) $$f_{Ld}=1-\frac{\delta }{h}$$

and for the pressure-induced flow as

(16) $$f_{Lp}=1+\left({\left(\frac{\delta }{h}\right)}^{3}\frac{e}{b}\frac{\eta }{\eta_{\delta }}+\frac{\left(1+\frac{e}{b}\right)\left[a+\frac{1+e/b}{\tan^{2}\varphi }\right]}{1+\frac{\eta_{\delta }}{\eta }{\left(\frac{h}{\delta }\right)}^{3}\frac{e}{b}}\right),$$

where δ is the flight clearance between the flight and the barrel, and *e* is the flight width.

Combined, the finalized model for the volumetric throughput in the melt conveying zone is

(17) $$\dot{V}=\frac{v_{0z}ibh}{2}{f_{d}f_{Ld}f}_{\eta d}-\frac{ibh^{3}}{12\eta }\frac{\Delta p}{Z}f_{p}f_{LP}f_{\eta p}$$

The equation is now solved for Δp as it is the only unknown variable in the system; the rest is derived from the extrusion experiments, the geometry of the screw (Table 2), and the results of the rheological model in Equation (1). By setting the pressure at the tip of the screw equal to the pressure measured in the extrusion experiment, the model can be used to iteratively calculate the pressure in the metering zone backward from the screw tip (25 D) to the beginning of the melt conveying zone (16 D). The calculated pressure distribution can then be compared to the pressure measured in the extrusion experiments to confirm the accuracy of the simulation.

A potential issue with the explained simulation is the fact that the perpendicular flow in the channel is barely addressed. Therefore, the method of using a representative viscosity, as formulated by Giesekus and Langer [37], is incorporated into the simulation model. The idea is that there exists a point within the shear rate profile of a fluid, where shear thinning and Newtonian behavior yield the exact same shear rate at a given volume flow rate and shear stress. In this way, Newtonian equations can be used to calculate values found in shear-thinning fluids [38]. The representative shear rate can be found in a rectangular profile at the representative height $h_{s}$ and the correction factor $e_{◻}$ with

(18) $$h_{s}=\frac{h}{2}\cdot e_{◻}\mathrm{with}\;e_{◻}={\left(\frac{3}{2+n}\right)}^{\frac{1}{n+1}}.$$

One can now use $h_{s}$ to calculate the representative shear rate ${\dot{\gamma }}_{rep}$. To be more precise, we look at the representative shear rate in both longitudinal (${\dot{\gamma }}_{z_{rep}}$) and perpendicular (${\dot{\gamma }}_{x_{rep}}$) flow directions within the channel. The shear rates are further split into drag-induced and pressure-induced parts, which can now be calculated with equations fit for Newtonian fluids, such as

(19) $${\dot{\gamma }}_{{z,d}_{rep}}=\frac{v_{0z}}{h_{s}}$$

for the drag-induced representative shear rate in channel direction ${\dot{\gamma }}_{{z,d}_{rep}}$, or

(20) $${\dot{\gamma }}_{{z,p}_{rep}}=\frac{{6\cdot \dot{V}}_{z,p}}{b\cdot {h_{s}}^{2}}\cdot e_{◻}$$

for the pressure-induced representative shear rate in channel direction ${\dot{\gamma }}_{{z,p}_{rep}}$. Here, ${\dot{V}}_{z,p}$ is the pressure-induced volumetric throughput. Perpendicular to the channel, the equations used are as follows:

(21) $${\dot{\gamma }}_{{x,s}_{rep}}=\frac{v_{0x}}{h_{s}}$$

for the drag-induced representative shear rate perpendicular to the channel direction and

(22) $${\dot{\gamma }}_{{x,p}_{rep}}=\frac{3v_{0x}}{h_{s}}\cdot e_{◻}$$

for the pressure-induced representative shear rate perpendicular to the channel direction. The separated drag- and pressure-induced parts are simply added together, as follows:

(23) $${\dot{\gamma }}_{z_{rep}}={\dot{\gamma }}_{{z,d}_{rep}}+{\dot{\gamma }}_{{z,p}_{rep}};$$

(24) $${\dot{\gamma }}_{x_{rep}}={\dot{\gamma }}_{{x,d}_{rep}}+{\dot{\gamma }}_{{x,p}_{rep}}$$

and combined to the total representative shear rate using vectorial addition:

(25) $${\dot{\gamma }}_{rep}=\sqrt{{{\dot{\gamma }}_{x_{rep}}}^{2}+{{\dot{\gamma }}_{z_{rep}}}^{2}}.$$

The representative viscosity $\eta_{rep}$ can then be computed using a rheological model like Bird–Carreau–Yasuda [35] and substituting the standard shear rate $\dot{\gamma }$ with ${\dot{\gamma }}_{rep}$.

The simulation model presented in Equation (17) is now adapted to include the newly calculated representative viscosity. As such, the influence of perpendicular flow in the channel is now taken into account in the representative viscosity. The final model used for the estimation of the volumetric throughput in the melt conveying zone is given in Equation (26):

(26) $$\dot{V}=\frac{v_{0z}ibh}{2}{f_{d}f_{Ld}f}_{\eta d}-\frac{ibh^{3}}{12\eta_{rep}}\frac{\Delta p}{Z}f_{p}f_{LP}f_{\eta p}.$$

Both the basic model in Equation (17) and a representative model in Equation (26) are isothermal, as the melt temperature in the melt conveying zone is treated as a single value for the calculation of viscosity in the simulation.

## 3. Results

The parameters for the rheological fits for all mixtures can be found in Table 3, alongside the deviation of the fit from the original capillary rheometry data. Each mixture, apart from the pure materials, was fitted from direct measurement data, as well as data computed using mixing rules.

The average mass throughput of five measurements at certain screw speeds, the average melt temperature in the melt conveying zone during extrusion, and the melt density derived from melt volume rate (MVR) measurements for each mixture tested are listed in Table 4. The mass temperature is taken as the mean of the values measured from the mass temperature sensors at 16 D and 24 D, which are close to the set barrel temperature in the metering zone of 230 °C throughout the experiment. As such, the MVR measurements were conducted at 230 °C to receive melt density values close to the actual melt temperature during the experiment.

**Figure 3:**
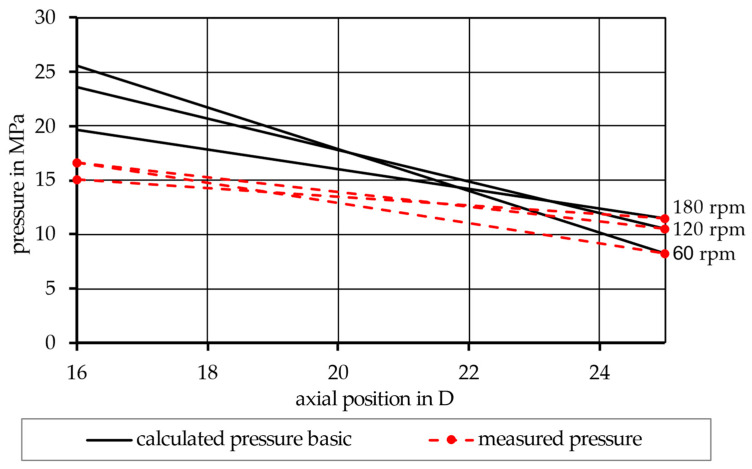
Comparison between the calculated and measured pressure distribution along the melt conveying zone for the miscible mixture 90PP_1_/10PP_2_, using the basic model.

**Figure 4:**
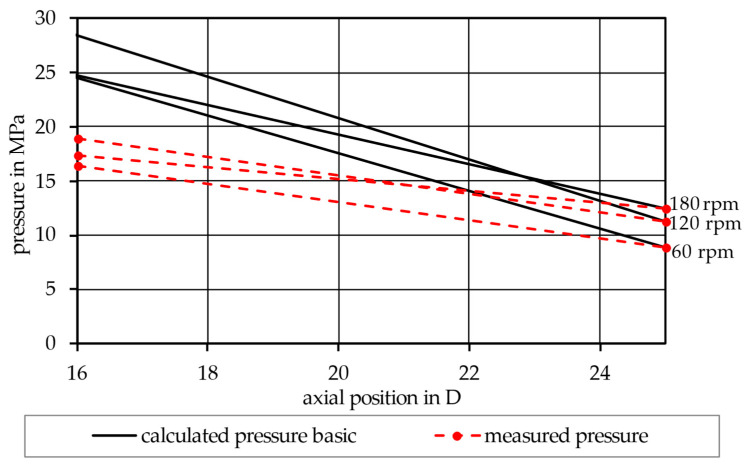
Comparison between the calculated and measured pressure distribution along the melt conveying zone for the immiscible mixture 90PP_1_/PPgMAH/10PA12, using the basic model.

The simulation was first performed according to the basic model in Equation (17), using the material input data from the rheological fits (Table 3) and the extrusion experiments (Table 4), as well as the dimensions of the screw (Table 2). This led to a pressure distribution within the melt conveying zone for an exemplary miscible mixture (90PP_1_/10PP_2_), as seen in Figure 3, and for an exemplary immiscible mixture (90PP_1_/PPgMAH/10PA12), as seen in Figure 4. Looking at the red graphs, which show the pressure values measured by the pressure transducers at the beginning of the melt conveying zone and at the screw tip, a pressure drop throughout the zone can be observed. By increasing the speed of the screw, an increase in pressure at the tip of the screw can be observed. Consequently, the pressure also rises at the beginning of the zone; however, for both mixtures depicted, as well as for the other mixtures subjected to the experiment, increasing the screw rotations from 120 to 180 rpm did not increase the pressure measured at an axial position of 16 D at the same rate. This led to lower absolute pressure values at this position, resulting in a lower pressure drop in the zone. The black graphs depict the calculated pressure values at the given screw speeds. Here, it can be seen that, when using the basic model in Equation (17), the pressure drop is vastly overestimated. Nevertheless, the previously described effect of a lower pressure drop at 180 rpm is simulated identically to the measured values in a qualitative way.

**Figure 5:**
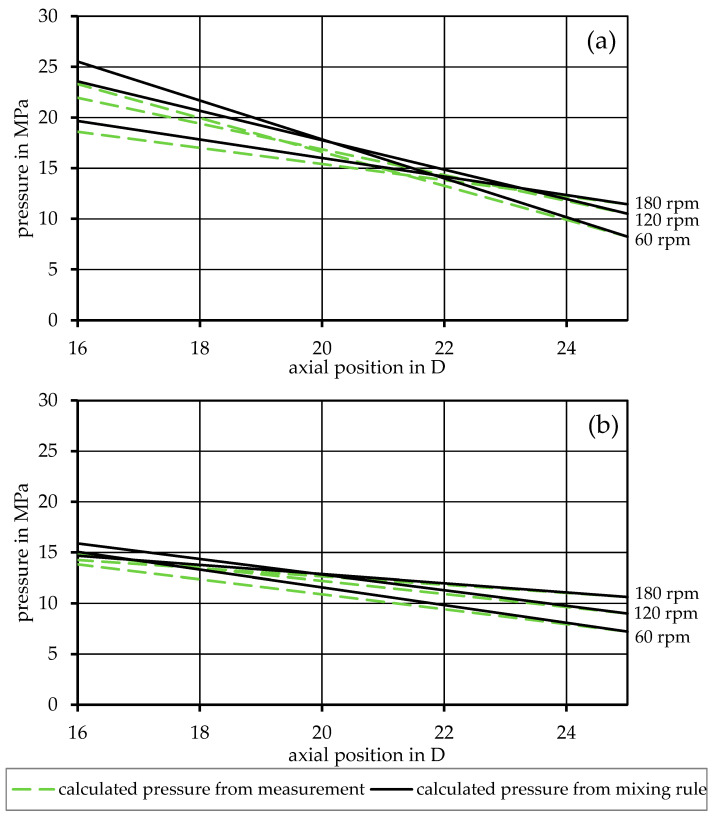
Comparison between rheological input data derived from measurements and rheological input data calculated from mixing rules in the basic model for the mixtures (**a**) 90PP_1_/10PP_2_ and (**b**) 50PP_1_/50PP_2_.

**Figure 6:**
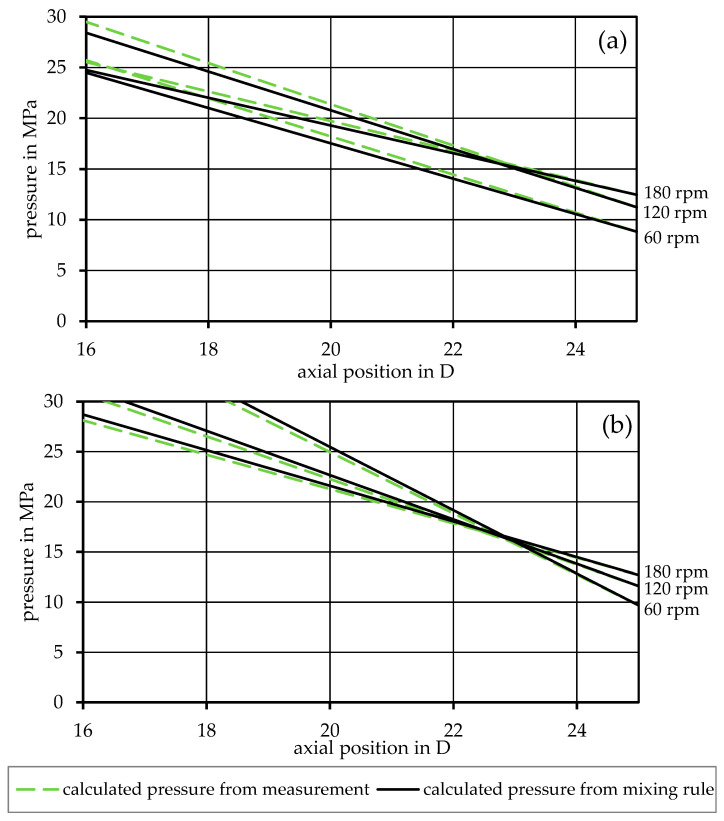
Comparison between rheological input data derived from measurements and rheological input data calculated from mixing rules in the basic model for the mixtures (**a**) 90PP_1_/PPgMAH/10PA12 and (**b**) 80PP_1_/PPgMAH/20PA12.

To confirm the validity of substituting the rheological input data from measurements with data from mixing rules, the calculation was performed for the same mixture with both sets of input data. The results are exemplary, as shown in Figure 5 for two of the miscible mixtures and in Figure 6 for two of the immiscible ones. Here, the black graphs show the results of the simulation using mixing rules compared to the gray graphs, which are calculated using the measured viscosity values. The graphs show good agreement, especially for the immiscible blends.

**Figure 7:**
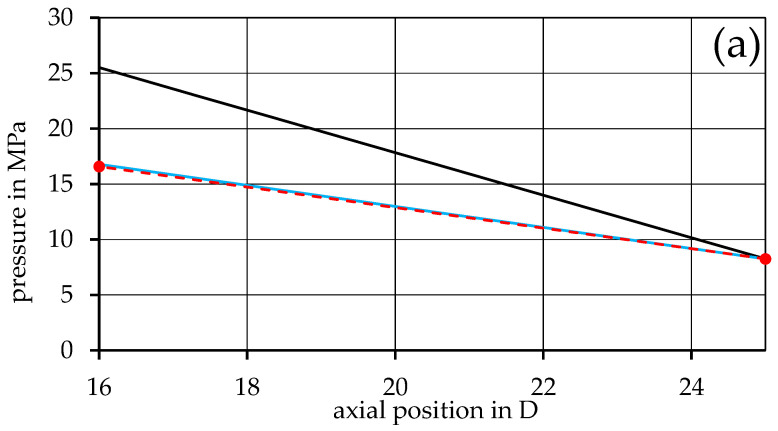

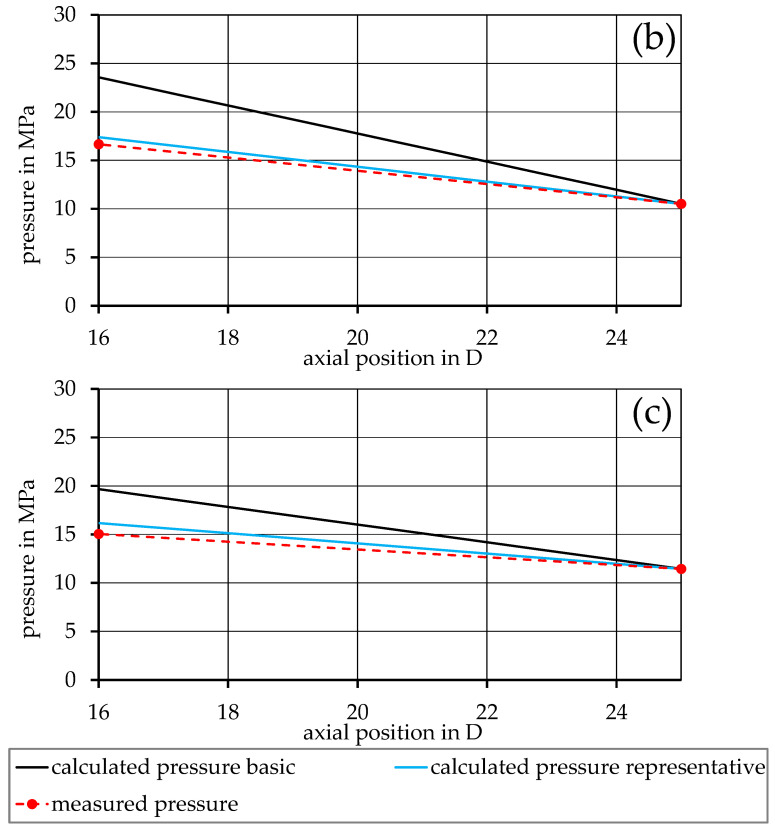
Comparison of the pressure distribution calculated from the basic model, the model with implemented representative viscosity, and the measured values of the miscible mixture 90PP_1_/10PP_2_ for the screw speeds (**a**) 60 rpm, (**b**) 120 rpm, and (**c**) 180 rpm.

**Figure 8:**
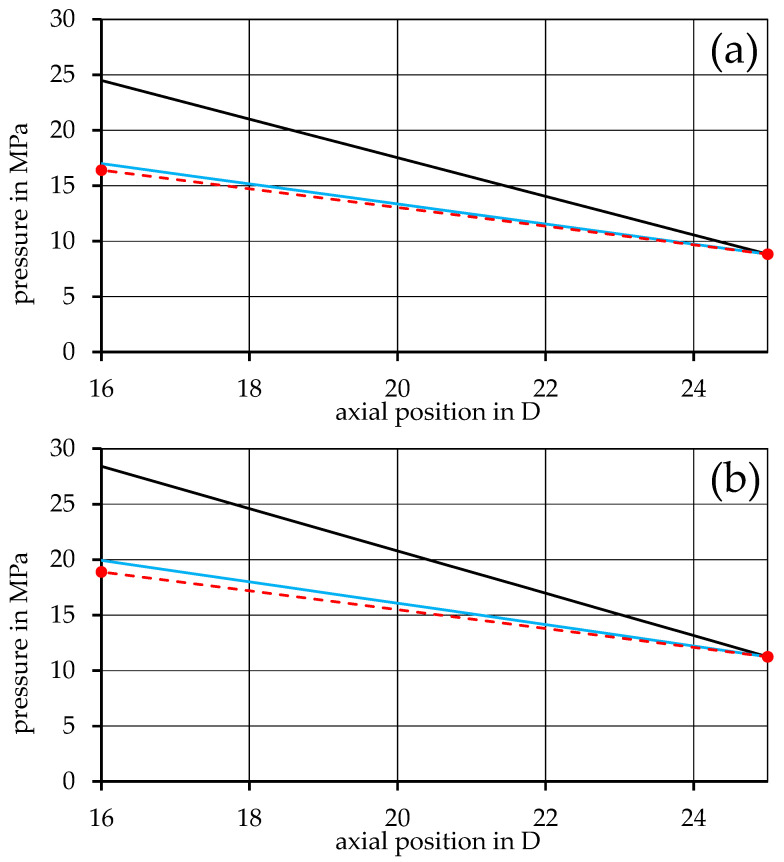

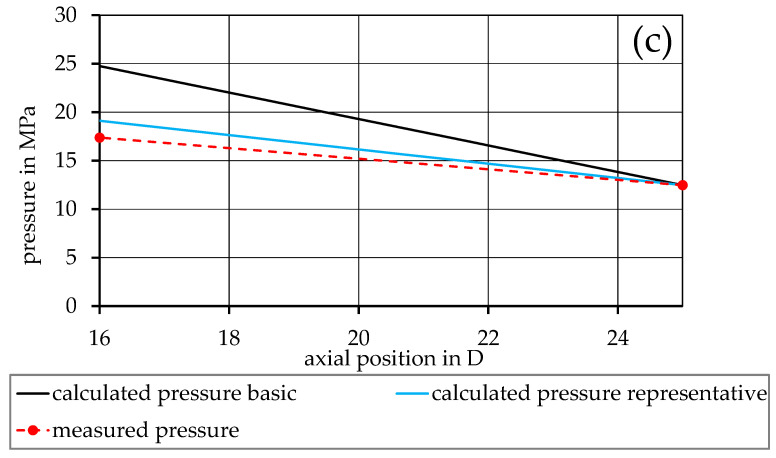
Comparison of the pressure distribution calculated from the basic model, the model with implemented representative viscosity, and the measured values of the immiscible mixture 90PP_1_/PPgMAH/10PA12 for the screw speeds (**a**) 60 rpm, (**b**) 120 rpm, and (**c**) 180 rpm.

From this point on, only the rheological input data derived from mixing rules is used for the simulation. By introducing the method of representative viscosity into the simulation, as shown in Equation (26), the perpendicular flow in the channel can be considered. A direct comparison with the previous model is shown in Figure 7 and Figure 8 for the 90PP_1_/10PP_2_ and 90PP_1_/PPgMAH/10PA12 mixtures, respectively, as a representative of the other mixtures. For all mixtures, a drastic increase in the accuracy of the simulation can be seen, with the blue graph representing the refined representative model, closely following the measurement values. The simulation is most accurate at the lowest screw speed considered, with a slightly increasing deviation from the extrusion measurements at higher speeds.

## 4. Discussion

Looking at Figure 3 and Figure 4, an overestimation of the pressure drop can be observed in the results of the basic simulation model. The most likely cause of this (and the reason why the representative model was introduced later on) lies in the disregard for the perpendicular flow within the channel. As Rauwendaal proposes, the total volumetric flow within the melt conveying zone can be considered as the result of drag and pressure-induced flow, as given by Equations (8)–(10) [3]. However, only the melt flow in the channel direction is taken into account, as only the term for melt velocity in the channel direction $v_{0z}$ is part of the basic model. The perpendicular flow inside the channel can, to a certain extent, obstruct the flow in the channel direction [4,19,38]. Thus, the basic model tends to exaggerate the viscosity of the system, and this in turn leads to an overshoot in the calculated pressure distribution. This is then remedied in the representative model by considering both directional parts of the melt velocity, $v_{0z}$ and $v_{0x}$, during the calculation of the representative shear rate used to receive the representative shear viscosity.

Regarding the differences between rheological input data for the calculations that come from direct measurement data or are created using mixing rules, as shown in Figure 5 and Figure 6, the propagation of errors must be discussed. When relying on the mixing rules to create the rheological fits for the mixtures, reliable measurement data for the base materials are of high importance. As these data sets are used as the baseline for the entire simulation, any errors found here will affect all further calculations and may result in deviations that can further propagate and lead to undesired results. Nevertheless, Figure 7 and Figure 8, in which the data used for the simulation are based on the mixing rules for the rheological part, show desirable simulation results with appropriate accuracy, even with deviations of the material fits, as listed in Table 3.

**Figure 9:**
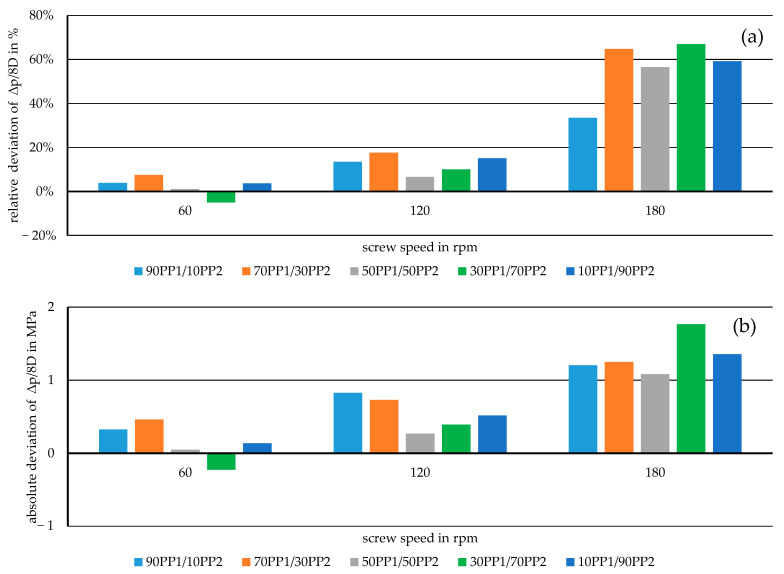
Relative (**a**) and absolute (**b**) deviations from the measured data of the pressure drop in the melt conveying zone over 8 D as simulated through the representative model for miscible mixtures.

**Figure 10:**
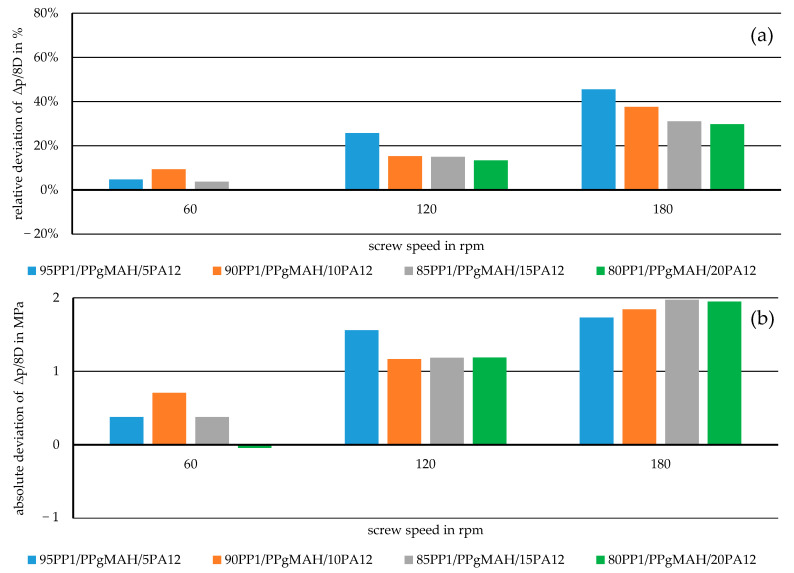
Relative (**a**) and absolute (**b**) deviations from the measured data of the pressure drop in the melt conveying zone over 8 D as simulated through the representative model for immiscible mixtures.

For a better overview of the accuracy of the representative model compared to the measured values, the deviations for the mixtures are further investigated. For this, the pressure drop *∆p* within the melt conveying zone over an axial distance of 8 D is calculated through the representative model, and its relative and absolute deviations from the measured pressure drop over the same distance are computed. This specific axial distance of 8D was chosen because it coincides with the distance between the pressure transducers mounted onto the lab-scale extruder at 16 D and 24 D. The results for all the mixtures subjected to the experiment, aside from the pure materials at different screw speeds, are shown in Figure 9 and Figure 10.

From these deviations, the decrease in accuracy of the model with increasing screw speed can be seen. While shifting from the basic model for the initial calculations to the representative model certainly lowered these deviations considerably, it is still an isothermal simulation model. With an increase in the screw speed in the system, the material in the channel is subjected to higher shear-based temperature increases [4]. This may result in lower viscosity of the polymer mixtures [21], which in turn leads to a lower actual pressure drop over the length of the melt conveying zone. Another aspect to consider when looking at the higher inaccuracies at rising screw speeds is the sampling rate of the pressure transducers. As the material and the screw flight pass by the sensor faster, the measured pressure values fluctuate more over the course of the 15 min during which the materials are extruded. This may indicate that the measured data used for comparison at 180 rpm were not as precise as the values at 60 rpm or even 120 rpm. Therefore, some of the deviations may come from the experiment rather than from the simulation model itself.

Implementing a way to also consider mass temperature changes in the simulation, for example, using the method of equivalent viscosity [38], is planned for future work. In this way, heating effects through changes in shear rate can be taken into account, which should yield even more accurate predictions of the pressure distribution in the extruder zone, especially at higher screw speeds.

## 5. Conclusions

In this study, a new approach to simulating the melt conveying zone of a single-screw extruder for polymer mixtures that reduces the rheological measurements needed for the simulation was presented. Using mixing rules, the rheological properties of various polymer blends, created from two PP grades and from a compatibilized PP grade and PA12, were approximated before being subjected to two variations of isothermal analytical flat plate simulation models. To verify the reliability of the proposed model, the polymer mixtures studied were also tested in extrusion experiments in a lab-scale extruder equipped with pressure and temperature sensors, and the results of the simulation and extrusion experiments were compared.

The main conclusions of this paper are that, for both miscible and immiscible polymer mixtures, using mixing rules to replace direct rheological measurement data is a viable strategy to apply in the simulation of recycling processes, where mixtures are more prevalent. In this way, the time needed for extensive measurements of various mixtures with different weight fractions of their components can be saved, while still yielding comparable results when predicting an extrusion process through simulation.

While the basic simulation model shown here overestimates the actual pressure drop measured during the experiments, its adaptation by further implementing the method of representative viscosity provides a promising way to compute the pressure distribution in the melt conveying zone, with relative deviations of the pressure drop lower than 5% for certain mixtures at low screw speeds, which translates to absolute deviations around 0.5 MPa.

## Figures and Tables

**Table 1:** Blends of PP_1_, PP_2_, PA12, and PPgMAH used.

Base Polymer	Concentration Base Polymer (wt%)	Contaminating Polymer	Concentration Contaminating Polymer (wt%)
PP_1_	100	-	-
PP_1_	90	PP_2_	10
PP_1_	70	PP_2_	30
PP_1_	50	PP_2_	50
PP_1_	30	PP_2_	70
PP_1_	10	PP_2_	90
PP_2_	100	-	-
PP_1_	96	PPgMAH	4
PP_1_/PPgMAH *	95	PA12	5
PP_1_/PPgMAH *	90	PA12	10
PP_1_/PPgMAH *	85	PA12	15
PP_1_/PPgMAH *	80	PA12	20
PA12	100	-	-

* Mixtures containing PA12 were created using a premade masterbatch of PP_1_ containing 4 wt% PPgMAH.

**Table 2:** Dimensions of the screw used in the extrusion experiments.

Geometric Aspect	Screw Dimensions
Diameter (mm)	20
Length (L/D)	25
Pitch (L/D)	1
Flight width (mm)	2.5
Flight clearance (mm)	0.015
Feeding zone	
Length (L/D)	10
Channel depth (mm)	4
Compression zone	
Length (L/D)	6
Channel depth ratio	3.64
Melt conveying zone	
Length (L/D)	9
Channel depth (mm)	1.1

**Table 3:** Fitting parameters for the Bird–Carreau–Yasuda model. The parameters for all the mixtures are found through a numerical solver.

Mixture	Source	η_0_ (Pa s)	B (s)	n	a	E_0_ (J/mol)	T_B_ (K)	Deviation
100PP_1_	Measurement	12,596.904	0.198	0.1666	0.445	37,311.25	473.150	0.019
90PP_1_/10PP_2_	Measurement	11,583.003	0.168	0.1583	0.427	37,946.07	473.150	0.019
	Mixing Rule	5882.389	0.111	0.1876	0.539	39,869.22	473.150	0.015
70PP_1_/300PP_2_	Measurement	6843.851	0.073	0.1271	0.426	34,920.43	473.150	0.025
	Mixing Rule	5309.360	0.105	0.1980	0.550	42,576.17	473.150	0.017
50PP_1_/50PP_2_	Measurement	5187.324	0.077	0.1642	0.474	36,661.24	473.150	0.018
	Mixing Rule	5543.623	0.073	0.1577	0.435	39,483.40	473.150	0.011
30PP_1_/70PP_2_	Measurement	4247.685	0.055	0.1485	0.444	35,361.64	473.150	0.015
	Mixing Rule	2961.148	0.039	0.1645	0.485	42,216.90	473.150	0.019
10PP_1_/90PP_2_	Measurement	2470.133	0.029	0.1325	0.483	36,652.37	473.150	0.012
	Mixing Rule	2016.089	0.019	0.1091	0.482	41,503.27	473.150	0.027
100PP_2_	Measurement	2047.812	0.020	0.1058	0.479	37,212.15	473.150	0.019
100PP_1_/PPgMAH	Measurement	15,939.316	0.05	0.0125	0.264	47,166.06	503.150	0.023
95PP_1_/PPgMAH/05PA12	Measurement	4129.580	0.063	0.1666	0.501	38,160.74	503.150	0.019
	Mixing Rule	14,153.343	0.048	0.0114	0.273	38,853.59	503.150	0.013
90PP_1_/PPgMAH/10PA12	Measurement	4215.396	0.062	0.1694	0.499	37,977.38	503.150	0.019
	Mixing Rule	16,993.074	0.057	0.0125	0.280	66,253.97	503.150	0.011
85PP_1_/PPgMAH/15PA12	Measurement	4465.000	0.063	0.1676	0.487	37,431.24	503.150	0.018
	Mixing Rule	22,459.861	0.072	0.0274	0.250	37,121.16	503.150	0.013
80PP_1_/PPgMAH/20PA12	Measurement	5366.491	0.073	0.1713	0.457	40,986.60	503.150	0.016
	Mixing Rule	27,675.946	0.093	0.0366	0.246	50,773.06	503.150	0.013
100PA12	Measurement	4217.026	0.030	0.3293	0.622	50,157.67	503.150	0.106

**Table 4:** Mass throughput and mass temperature values at given screw speeds. The melt density values listed are taken from MVR measurements, which were performed at 230 °C.

Mixture	Screw Speed (rpm)	Mass Throughput (kg/h)	Mass Temperature (°C)	Melt Density at 230 °C (kg/m^3^)
100PP_1_	60	1.549	229.290	740
	120	2.694	228.725	740
	180	3.928	228.548	740
90PP_1_/10PP_2_	60	1.560	228.774	740
	120	2.845	228.064	740
	180	3.997	227.371	740
70PP_1_/300PP_2_	60	1.494	228.274	740
	120	2.741	228.161	740
	180	3.904	227.612	740
50PP_1_/50PP_2_	60	1.435	228.967	740
	120	2.727	228.096	740
	180	3.912	228.338	740
30PP_1_/70PP_2_	60	1.441	227.741	740
	120	2.839	229.467	740
	180	4.081	230.016	740
10PP_1_/90PP_2_	60	1.495	227.887	740
	120	2.777	229.209	740
	180	4.057	229.483	740
100PP_2_	60	1.450	229.338	740
	120	2.651	228.242	740
	180	3.982	228.693	740
100PP_1_/PPgMAH	60	1.540	228.612	741
	120	2.773	228.322	741
	180	3.943	228.532	741
95PP_1_/PPgMAH/05PA12	60	1.561	229.290	746
	120	2.919	229.596	746
	180	4.097	229.387	746
90PP_1_/PPgMAH/10PA12	60	1.558	230.629	753
	120	3.010	229.129	753
	180	4.221	231.903	753
85PP_1_/PPgMAH/15PA12	60	1.659	228.645	758
	120	3.030	227.709	758
	180	4.369	228.677	758
80PP_1_/PPgMAH/20PA12	60	1.783	230.003	764
	120	3.099	228.766	764
	180	4.390	230.383	764

## Data Availability

The original contributions presented in this study are included in the article; further inquiries can be directed to the corresponding author.

## Appendix A

**Table A1:** Measured viscosity data of the pure PP types at measurement temperatures of 200 and 230 °C.

$\dot{\gamma }$ PP1 200 °C	η PP1 200 °C	$\dot{\gamma }$ PP2 200 °C	η PP2 200 °C	$\dot{\gamma }$ PP1 230 °C	η PP1 230 °C	$\dot{\gamma }$ PP2 230 °C	η PP2 230 °C
11.16	2232.93	10.51	1054.75	11.04	1957.08	10.36	628.91
28.65	1284.30	24.67	757.27	27.91	1183.19	23.79	518.47
66.05	783.48	56.13	530.42	64.21	735.00	54.11	392.09
153.17	452.61	130.53	349.94	146.25	441.12	123.19	275.21
341.96	260.99	308.89	208.92	331.14	258.06	285.65	178.33
802.71	142.49	736.65	116.15	756.67	145.96	684.26	104.17
1964.28	70.55	1742.50	61.83	1770.23	78.27	1640.65	56.40
4413.11	36.85	4085.47	32.17	4418.68	37.98	3822.34	30.13
9667.29	19.81	9461.38	16.51	11,241.05	17.17	9015.37	15.54
16,904.08	13.21	16,742.36	10.91	19,808.35	10.93	16,416.06	10.04

**Table A2:** Measured viscosity data of PP1/PPgMAH and PA12 at 230 and 250 °C.

$\dot{\gamma }$ PP1/PPgMAH 230 °C	η PP1/PPgMAH 230 °C	$\dot{\gamma }$ PA12 230 °C	η PA12 230 °C	$\dot{\gamma }$ PP1/PPgMAH 250 °C	η PP1/PPgMAH 250 °C	$\dot{\gamma }$ PA12 250 °C	η PA12 250 °C
10.38	1412.35	19.58	1585.16	10.80	1189.36	8.24	2148.32
25.99	1064.44	46.85	1523.74	25.58	814.29	17.71	1757.46
61.74	652.26	108.22	1261.41	58.83	562.47	38.64	1385.98
142.06	401.29	251.82	899.55	138.02	347.63	84.95	1065.02
329.13	231.52	603.50	565.40	320.93	203.55	190.67	765.44
757.00	130.03	1478.09	307.94	733.86	116.28	441.92	499.65
1762.59	70.03	2957.63	195.87	1676.36	64.87	1071.40	285.17
4144.25	36.18	5849.49	145.42	4007.33	33.68	2712.68	143.32
12,936.16	13.75			10,003.69	16.01	6487.90	71.19
36,861.17	5.43			18,172.23	10.08		

**Table A3:** Measured viscosity data of the PP1/PP2 mixtures at 200 °C.

$\dot{\gamma }$ 90PP1/10PP2 200 °C	η 90PP1/10PP2 200 °C	$\dot{\gamma }$ 70PP1/30PP2 200 °C	η 70PP1/30PP2 200 °C	$\dot{\gamma }$ 50PP1/50PP2 200 °C	η 50PP1/50PP2 200 °C	$\dot{\gamma }$ 30PP1/70PP2 200 °C	η 30PP1/70PP2 200 °C	$\dot{\gamma }$ 10PP1/90PP2 200 °C	η 10PP1/90PP2 200 °C
22.88	1371.27	11.08	2036.03	10.99	1611.33	22.30	975.10	22.31	794.66
62.20	765.16	29.18	1167.16	25.71	1066.53	57.64	621.41	55.52	568.03
147.80	443.14	65.29	710.51	60.79	723.14	135.50	387.95	133.94	355.43
337.98	253.31	147.19	434.26	144.73	418.97	316.94	228.79	311.27	210.94
776.74	141.21	337.98	247.99	331.32	243.59	738.55	128.72	734.70	119.15
1891.64	71.79	752.22	142.51	737.86	140.87	1651.40	72.84	1700.51	64.73
4404.70	36.31	1803.28	75.34	1760.32	75.57	3908.87	38.75	3835.44	35.59
12,867.65	14.71	4386.00	36.87	4342.20	36.75	9402.64	19.19	9026.72	18.40
		9910.65	19.08	10,085.44	18.48	16,691.30	12.67	16,470.85	11.85
		17,179.09	12.78	17,565.97	12.24				

**Table A4:** Measured viscosity data of the PP1/PP2 mixtures at 230 °C.

$\dot{\gamma }$ 90PP1/10PP2 230 °C	η 90PP1/10PP2 230 °C	$\dot{\gamma }$ 70PP1/30PP2 230 °C	η 70PP1/30PP2 230 °C	$\dot{\gamma }$ 50PP1/50PP2 230 °C	η 50PP1/50PP2 230 °C	$\dot{\gamma }$ 30PP1/70PP2 230 °C	η 30PP1/70PP2 230 °C	$\dot{\gamma }$ 10PP1/90PP2 230 °C	η 10PP1/90PP2 230 °C
10.88	1412.30	10.71	1234.29	10.74	1096.01	10.33	745.83	10.20	574.38
26.25	923.46	25.25	869.82	24.92	772.13	23.80	619.60	23.18	506.79
60.57	607.76	59.06	589.92	57.24	547.73	53.77	465.84	52.84	396.95
140.42	372.61	138.58	362.04	134.65	346.85	126.85	318.33	122.06	280.86
323.74	218.22	319.38	213.53	310.95	207.23	301.58	192.19	288.14	179.64
772.77	119.07	736.61	121.57	722.21	119.09	710.95	109.83	692.25	103.30
1767.91	64.04	1708.62	66.39	1648.27	66.48	1626.89	61.58	1615.21	57.29
4077.20	34.11	3977.52	35.10	3791.35	36.30	3753.18	33.68	3732.45	31.27
9543.84	17.27	9722.46	17.21	9331.12	17.92	8834.44	17.44	8854.92	16.12
16,823.99	11.46	17,843.72	10.77	17,303.28	11.19	16,007.48	11.42	16,172.53	10.44

**Table A5:** Measured viscosity data of the PP1/PPgMAH/PA12 mixtures at 230 °C.

$\dot{\gamma }$ 95PP1/PPgMAH/5PA12 230 °C	η 95PP1/PPgMAH/5PA12 230 °C	$\dot{\gamma }$ 90PP1/PPgMAH/10PA12 230 °C	η 90PP1/PPgMAH/10PA12 230 °C	$\dot{\gamma }$ 85PP1/PPgMAH/15PA12 230 °C	η 85PP1/PPgMAH/15PA12 230 °C	$\dot{\gamma }$ 80PP1/PPgMAH/20PA12 230 °C	η 80PP1/PPgMAH/20PA12 230 °C
10.97	1564.79	10.64	1712.37	11.07	1691.22	10.99	1807.83
26.95	993.20	26.36	1192.64	26.70	1063.02	27.52	1119.55
61.29	648.47	62.53	740.10	61.57	701.99	62.70	710.60
142.01	395.85	144.59	444.09	143.60	418.52	142.68	435.84
327.63	229.52	333.45	255.71	330.80	242.57	332.36	250.91
746.47	130.55	781.32	139.96	770.16	134.08	773.87	137.79
1742.62	70.70	1852.57	72.75	1773.18	72.34	1691.48	78.38
4183.34	35.84	4339.74	37.12	4056.73	38.54	3752.81	44.31
11,361.95	15.52	11,601.36	16.27	9610.83	19.42	12,319.38	16.63
22,425.61	8.66	22,980.69	9.02	17,343.03	12.45		

**Table A6:** Measured viscosity data of the PP1/PPgMAH/PA12 mixtures at 250 °C.

$\dot{\gamma }$ 95PP1/PPgMAH/5PA12 250 °C	η 95PP1/PPgMAH/5PA12 250 °C	$\dot{\gamma }$ 90PP1/PPgMAH/10PA12 250 °C	η 90PP1/PPgMAH/10PA12 250 °C	$\dot{\gamma }$ 85PP1/PPgMAH/15PA12 250 °C	η 85PP1/PPgMAH/15PA12 250 °C	$\dot{\gamma }$ 80PP1/PPgMAH/20PA12 250 °C	η 80PP1/PPgMAH/20PA12 250 °C
10.93	1322.41	11.02	1383.71	11.00	1451.30	10.96	1438.56
26.67	854.35	26.82	874.38	26.73	923.44	26.89	917.96
60.82	562.02	60.47	583.10	61.94	600.94	62.02	591.61
139.29	348.70	139.53	361.31	142.24	362.84	140.62	363.69
320.02	206.56	320.78	212.82	320.30	217.49	315.78	220.48
732.93	118.09	731.11	122.36	720.29	127.16	730.69	126.24
1693.41	65.09	1707.39	66.82	1718.32	68.64	1744.21	67.03
4024.87	33.77	4100.18	34.10	4242.49	33.72	4248.64	33.41
12,306.15	13.16	10,033.85	16.48	9871.78	16.98	9590.90	17.31
31,211.24	5.74	18,075.70	10.47	17,178.96	11.33		
